# A Characterization of the Compound Multiparameter Hermite Gamma Distribution via Gauss's Principle

**DOI:** 10.1155/2013/468418

**Published:** 2013-10-31

**Authors:** Werner Hürlimann

**Affiliations:** Wolters Kluwer Financial Services, Seefeldstrasse 69, 8008 Zürich, Switzerland

## Abstract

We consider the class of those distributions that satisfy Gauss's principle (the maximum likelihood estimator of the mean is the sample mean) and have a parameter orthogonal to the mean. It is shown that this so-called “mean orthogonal class” is closed under convolution. A previous characterization of the compound gamma characterization of random sums is revisited and clarified. A new characterization of the compound distribution with multiparameter Hermite count distribution and gamma severity distribution is obtained.

## 1. Introduction

The topic of maximum likelihood characterizations of distributions has a long history and is an active field of contemporary mathematical sciences. It concerns the characterization of a (class of) probability distribution(s) through the structure of the maximum likelihood estimator (MLE) of one or more parameters of interest (e.g., location, scale, etc.). Starting point is a famous result by Gauss [1] on the foundation of least squares theory (see, e.g., [2], [3, afterword, pages 208 and 215]). Given is a location family with continuous derivative. If the maximum likelihood estimator of the location parameter is the sample mean, then the distribution is normal. This important result has been discussed by Poincaré [4, Chapter 10], Teicher [5], Ferguson [6, 7], Marshall and Olkin [8], Bondesson [9], and Azzalini and Genton [10], by many other authors. The property that the MLE of the mean is the sample mean has been called *Gauss's principle* by Campbell [11] (see also [12, 13] and references therein). A brief account of the present contribution follows.

Within the framework of multiparameter distributions, consider the “mean orthogonal class” of those distributions that besides Gauss's principle have a parameterization such that the mean is orthogonal to some parameter vector, a property which can always be satisfied by Amari [14], Section 8. This class has been considered by Sprott [15]. A characterization of the mean orthogonal class through the cumulant generating function (cgf) has been formulated by Hürlimann [16]. Extending a result by Hudson [17], it is shown in Theorem 3 that this class is closed under convolution.  Section 3 is devoted to a characterization of random sums through the mean orthogonal class. Hürlimann [18] has established that the mean scaled severity in a compound model is necessarily gamma distributed provided that the count distribution and the distribution of the random sum belong to the mean orthogonal class, and some additional partial differential parameter equation can be solved. A followup to this construction for the individual model of risk theory is Hürlimann [19]. We clarify and simplify the original proof to obtain a characterization that is further used in Section 4. Based on a result by Puig and Valero [20], we derive in Theorem 17 a most stringent characterization, which allows compounding of the gamma distribution under a single count data family, namely, the multiparameter Hermite distribution. This one requires that the count distribution is closed under convolution and binomial subsampling.

## 2. Distributions with the Mean Orthogonal Property

Let *X* be a random variable whose distribution depends upon a vector (*μ*, *θ*) = (*μ*, *θ*_1_,…, *θ*_*m*_) of *m* + 1 parameters, where the mean *μ* is functionally independent of *θ*, that is, ∂*μ*/∂*θ*_*k*_ = 0, *k* = 1,…, *m*. The log likelihood of *X* is denoted by *ℓ*(*x*; *μ*, *θ*). We assume throughout that the cumulant generating function (cgf) *C*(*t*; *μ*, *θ*) = ln⁡{*E*[exp⁡(*tX*)]} exists and denotes the variance by *σ* ^2^ = *σ* ^2^(*μ*, *θ*). The standard regularity conditions for maximum likelihood estimation are supposed to hold. The vector **X** = (*X*_1_,…, *X*_*n*_) denotes a random sample of size *n*, which realizes the random variable *X*, and $\overset{-}{X}$ denotes the sample mean. We are interested in the class $\overset{-}{C}$ of distributions that satisfy Gauss's principle (the maximum likelihood estimator of the mean is the sample mean), that is, such that $\hat{\mu }=\overset{-}{X}$. A distribution belongs to this class if and only if there are functions *g*_*k*_ = *g*_*k*_(*μ*, *θ*), *k* = 1,…, *m*, *h* = *h*(*μ*, *θ*), such that the following equivalent partial differential equations hold (e.g., [15–17]):

(1) $$\begin{array}{c} \frac{\partial \ell \left(x;\mu ,\theta \right)}{\partial \mu }+\sum_{k=1}^{m}g_{k}\cdot \frac{\partial \ell \left(x;\mu ,\theta \right)}{\partial \theta_{k}}+h\cdot \left(x-\mu \right)=0,\\ \frac{\partial C}{\partial \mu }+\sum_{k=1}^{m}g_{k}\cdot \frac{\partial C}{\partial \theta_{k}}+h\cdot \left(\frac{\partial C}{\partial t}-\mu \right)=0. \end{array}$$

**Definition 1**

The mean *μ* is called *orthogonal* to the parameter vector *θ*, denoted by *μ*⊥*θ*, if one has *E*[(∂^2^ *ℓ*/∂*μ*∂*θ*_*k*_)] = *E*[(∂*ℓ*/∂*μ*)(∂*ℓ*/∂*θ*_*k*_)] = 0, *k* = 1,…, *m*.

The original motivation for parameter orthogonality is improvement of maximum likelihood estimation by reparameterization. In the class $\overset{-}{C}$ the number of maximum likelihood equations is reduced by one and parameter orthogonality decreases the often high correlation between the MLEs of the parameters since the MLEs of orthogonal parameters are asymptotically uncorrelated. Indeed, the expectations in (2) are elements of the (expected) Fisher information matrix, which determines the asymptotic covariance matrix of (*μ*, *θ*). In this respect, one is interested in the subclass ${\overset{-}{C}}^{⊥}$ of $\overset{-}{C}$ of all distributions satisfying besides $\hat{\mu }=\overset{-}{X}$ the *mean orthogonal property μ*⊥*θ*. This so-called *mean orthogonal class* is characterized as follows.

**Theorem 2 (Characterization of the mean orthogonal class)**

Let *X* be a random variable with cgf *C*(*t*; *μ*, *θ*) satisfying the above assumptions. Then, one has $X\in {\overset{-}{C}}^{⊥}$ if and only if the following quasi-linear partial differential equation is satisfied:

(2) $$\begin{array}{c} \sigma^{2}\cdot \frac{\partial C}{\partial \mu }-\left(\frac{\partial C}{\partial t}-\mu \right)=0. \end{array}$$

**Proof**

This is shown in Hürlimann [16].

Hudson [17, Theorem 1] has shown that the class $\overset{-}{C}$ is closed under convolution. In fact, convolution invariance holds under the more stringent mean orthogonal property.

**Theorem 3 (Convolution invariance of the mean orthogonal class)**

If $X_{1},X_{2}\in {\overset{-}{C}}^{⊥}$ are independent, then $X=X_{1}+X_{2}\in {\overset{-}{C}}^{⊥}$. More precisely, if $X_{i}\in {\overset{-}{C}}^{⊥}$ has cgf *C*_*i*_(*t*; *μ*_*i*_, *θ* ^(*i*)^), with *μ*_*i*_⊥*θ* ^(*i*)^, *i* = 1,2, then the cgf *C*(*t*; *μ*, *δ*, *θ*) of *X* = *X*_1_ + *X*_2_, with *μ* = *μ*_1_ + *μ*_2_, *δ* = |*μ*_1_ *σ*_2_^2^ − *μ*_2_ *σ*_1_^2^|, and *θ* = (*θ* ^(1)^, *θ* ^(2)^), satisfies (2) and one has $\hat{\mu }=\overset{-}{X}$, *μ*⊥(*δ*, *θ*).

**Proof**

Without loss of generality we assume that *δ* = *μ*_1_ *σ*_2_^2^ − *μ*_2_ *σ*_1_^2^ > 0. Since *μ* = *μ*_1_ + *μ*_2_ and *σ* ^2^ = *σ*_1_^2^ + *σ*_2_^2^, one can express (*μ*_1_, *μ*_2_) as a function of (*μ*, *δ*) through the parameter transformation *μ*_1_ = (*μσ*_1_^2^ + *δ*)/*σ* ^2^, *μ*_2_ = (*μσ*_2_^2^ − *δ*)/*σ* ^2^. Since $X_{1},X_{2}\in {\overset{-}{C}}^{⊥}$ and *C*(*t*; *μ*, *δ*, *θ*) = *C*_1_(*t*; *μ*_1_, *θ* ^(1)^) + *C*_2_(*t*; *μ*_2_, *θ* ^(2)^), one obtains

(3) σ2·∂C(t;μ,δ,θ)∂μ =σ2·∂μ1∂μ·∂C1(t;μ1,θ(1))∂μ1+σ2·∂μ2∂μ·∂C2(t;μ2,θ(2))∂μ2 =σ12·∂C1(t;μ1,θ(1))∂μ1+σ22·∂C2(t;μ2,θ(2))∂μ2 =∂C1(t;μ1,θ(1))∂t−μ1+∂C2(t;μ2,θ(2))∂t−μ2 =∂C(t;μ,δ,θ)∂t−μ,

which implies the result by (2) of Theorem 2.

**Example 4**

Binomial random variables and their convolutions belong to the class ${\overset{-}{C}}^{⊥}$. For two binomials this is shown in Hürlimann [16, Example 2] (see also [21]). For an arbitrary number of binomials this is derived in the Appendix of Hudson [17].

## 3. Mean Orthogonal Characterization of the Compound Gamma Distribution

Consider random sums of the type

(4) $$\begin{array}{c} X=\sum_{i=1}^{N}Y_{i}, \end{array}$$

where the *Y*_*i*_'s are independent and identically distributed nonnegative random variables, and *N* is a counting random variable defined on the nonnegative integers, which is independent of the *Y*_*i*_'s. The mean and variance of *X*, *N*, and *Y* ~ *Y*_*i*_ are denoted, respectively, by *μ*, *σ* ^2^, *μ*_*N*_, *σ*_*N*_^2^, and *μ*_*Y*_, *σ*_*Y*_^2^. The coefficient of variation of *X* is denoted by *κ* = *σ*/*μ*. In some applications, it is convenient to scale the severity *Y* by the mean *μ* such that the *mean scaled severity Z* = *Y*/*μ* ~ *Z*_*i*_ = *Y*_*i*_/*μ* has mean 1/*μ*_*N*_. The resulting sum

(5) $$\begin{array}{c} X=\mu \cdot \sum_{i=1}^{N}Z_{i} \end{array}$$

is called *mean scaled compound* random sum. The mean scaled compound model has important insurance risk applications. It has been studied in Hürlimann [18], which establishes that the mean scaled severity is necessarily gamma distributed provided that the random variables *N* and *X* belong to the mean orthogonal class and some additional partial differential parameter equation can be solved. A followup to this construction for the individual model of risk theory is Hürlimann [19]. We clarify and simplify the original proof to obtain a characterization of (4), which will be used in Section 4. In particular, (3.20) in Hürlimann [18] is not a consequence but an assumption. Since this equation is satisfied in the provided examples, this error does not harm the obtained result but must be rectified from a mathematical logical point of view. Also, the proof of Lemma 7 there will be simplified (proof of Lemma 8 below).

**Theorem 5 (Compound gamma characterization)**

Let $N\in {\overset{-}{C}}^{⊥}$ be a counting random variable with cgf *C*_*N*_(*t*; *p*, *θ*_*N*_). Suppose there exists a one-to-one coordinate transformation mapping (*p*, *θ*_*N*_) to (*μ*_*N*_, *θ*_*N*_) such that *μ*_*N*_ = *μ*_*N*_(*p*, *θ*_*N*_)⊥*θ*_*N*_, and set *λ*_*N*_ = − ln Pr (*N* = 0). Suppose the cgf *C*_*Y*_(*t*) of the severity *Y* exists, and let *C*(*t*) = *C*_*N*_(*C*_*Y*_(*t*)) be the cgf of the random sum *X* = ∑_*i*=1_^*N*^ *Y*_*i*_. Assume the cgf of the mean scaled severity *C*_*Z*_(*t*) = *C*_*Y*_(*t*/*μ*) is functionally independent of *μ*, and set *α* = (*μ*_*N*_ *κ* ^2^)^−1^, and *κ* = *σ*/*μ*. If $X\in {\overset{-}{C}}^{⊥}$ and *σ* ^2^ · (∂*λ*_*N*_/∂*μ*) = *μ*, then *Y* is gamma distributed with cgf *C*_*Y*_(*t*) = *α* · ln⁡{*α*/(*α* − *μ*_*Y*_ *t*)}.

To show this, some preliminaries are required. First, we review conditions under which $N\in {\overset{-}{C}}^{⊥}$. Given the probability generating function (pgf) *P*(*s*) = *E*[*s* ^*N*^] = ∑_*n*=0_^*∞*^ *p*(*n*)*s* ^*n*^ of *N*, it is very useful to consider the associated so-called *cumulant* pgf defined by

(6) G(s)=ln⁡{P(s)}+λN=∑k=1∞g(k)·sk,   λN=−ln⁡P(0)=G(1).

The given name stems from the following series representation of the cgf:

(7) CN(t)=ln⁡{P(et)}=G(et)−λN=∑k=1∞(ekt−1)·g(k).

**Remark 6**

The sequence *g*(*k*), *k* = 1,2,…, is the unique solution of the system of equations (e.g., [22, Corollary 2], [23], and [24, Theorem 1]):

(8) n·p(n)=∑k=1nk·g(k)·p(n−k),n=1,2,…, p(0)=e−λN.

If *g*(*k*) ≥ 0, *k* = 1,2,…, the distribution of *N* is compound Poisson with parameter *λ*_*N*_ and severity distribution *h*(*k*) = *g*(*k*)/*λ*_*N*_, *k* = 1,2,…. Otherwise, one speaks of the so-called *pseudo-compound Poisson* representation of the distribution.

**Lemma 7**

Let *N* be a counting random variable with cgf *C*_*N*_(*t*; *p*, *θ*_*N*_) of the form (7). Suppose there exists a one-to-one coordinate transformation mapping (*p*, *θ*_*N*_) to (*μ*_*N*_, *θ*_*N*_), and set *λ*_*N*_ = − ln Pr (*N* = 0). Then $N\in {\overset{-}{C}}^{⊥}$ with *μ*_*N*_ = *μ*_*N*_(*p*, *θ*_*N*_)⊥*θ*_*N*_ is equivalent to the following conditions:

(9) $$\begin{array}{c} \sigma_{N}^{2}\cdot \frac{\partial C_{N}\left(t\right)}{\partial \mu_{N}}=\frac{\partial C_{N}\left(t\right)}{\partial t}-\mu_{N}, \end{array}$$

(10) $$\begin{array}{c} \mu_{N}\cdot \frac{\partial G\left(s\right)}{\partial p}=\frac{\partial \lambda_{N}}{\partial p}\cdot s\cdot G^{\prime }\left(s\right), \end{array}$$

(11) $$\begin{array}{c} \mu_{N}\cdot \frac{\partial g\left(k\right)}{\partial p}=\frac{\partial \lambda_{N}}{\partial p}\cdot kg\left(k\right),\;k=1,2,\ldots . \end{array}$$

**Proof**

The condition (9) is a restatement of Theorem 2. Applying the chain rule of differential calculus, this condition transforms to

(12) $$\begin{array}{c} \sigma_{N}^{2}\cdot \frac{\partial C_{N}\left(t\right)}{\partial p}=\frac{\partial \mu_{N}}{\partial p}\cdot \left(\frac{\partial C_{N}\left(t\right)}{\partial t}-\mu_{N}\right). \end{array}$$

Now, by Lemma 8 below and the chain rule, one has

(13) $$\begin{array}{c} \sigma_{N}^{2}\cdot \frac{\partial \lambda_{N}}{\partial p}=\frac{\partial \mu_{N}}{\partial p}\cdot \mu_{N}. \end{array}$$

Inserting into (12) shows that

(14) $$\begin{array}{c} \mu_{N}\cdot \frac{\partial C_{N}\left(t\right)}{\partial p}=\frac{\partial \lambda_{N}}{\partial p}\cdot \left(\frac{\partial C_{N}\left(t\right)}{\partial t}-\mu_{N}\right). \end{array}$$

The statements (10) and (11) follow by using the representation (7).

**Lemma 8**

If $N\in {\overset{-}{C}}^{⊥}$, then the partial differential parameter equation *σ*_*N*_^2^ · (∂*λ*_*N*_/∂*μ*_*N*_) = *μ*_*N*_ holds.

**Proof**

The representation (7) implies that *λ*_*N*_ = *G*(1) = ∑_*k*=1_^*∞*^ *g*(*k*), *C*_*N*_′(0) = ∑_*k*=1_^*∞*^ *kg*(*k*) = *μ*_*N*_. Now, using (7) one sees that (9) is equivalent to *σ*_*N*_^2^ · (∂*g*(*k*)/∂*μ*_*N*_) = *kg*(*k*), *k* = 1,2,…. It follows that *σ*_*N*_^2^ · (∂*λ*_*N*_/∂*μ*_*N*_) = ∑_*k*=1_^*∞*^ *σ*_*N*_^2^ · (∂*g*(*k*)/∂*μ*_*N*_) = ∑_*k*=1_^*∞*^ *kg*(*k*) = *μ*_*N*_.

**Proof of Theorem 5**

Let *M*_*Y*_(*t*) = exp⁡{*C*_*Y*_(*t*)} be the moment generating function of *Y*. Expressed in terms of the mean scaled severity *Z* = *Y*/*μ* one has *M*_*Y*_(*t*) = *M*_*Z*_(*μt*). The relationship (7) for the cgf *C*_*N*_(*t*) yields the series expansion:

(15) $$\begin{array}{c} C\left(t\right)=C_{N}\left(\ln M_{Z}\left(\mu t\right)\right)=\sum_{k=1}^{\infty }g\left(k\right)\cdot M_{Z}^{k}\left(\mu t\right)-\lambda_{N}. \end{array}$$

By Theorem 2 one has $X\in {\overset{-}{C}}^{⊥}$ if and only if the equation *σ* ^2^ · (∂*C*/∂*μ*) − ((∂*C*/∂*t*) − *μ*) = 0 is satisfied. With the series representation for *C*(*t*) and the assumption *σ* ^2^ · (∂*λ*_*N*_/∂*μ*) = *μ*, this equation is equivalent to the following condition (use that *M*_*Z*_(*t*) does not depend on *μ*):

(16) (μ−σ2t)·MZ′(μt)·∑k=1∞kg(k)·MZk−1(μt)  =MZ(μt)·σ2·∑k=1∞∂g(k)∂μ·MZk−1(μt).

Now, by Lemma 7 and (10), one has the identity (use the differential chain rule)

(17) $$\begin{array}{c} \mu_{N}\cdot \frac{\partial g\left(k\right)}{\partial \mu }=\frac{\partial \lambda_{N}}{\partial \mu }\cdot kg\left(k\right), \end{array}$$

which, together with *σ* ^2^ · (∂*λ*_*N*_/∂*μ*) = *μ*, implies that

(18) $$\begin{array}{c} \sigma^{2}\cdot \frac{\partial g\left(k\right)}{\partial \mu }=\frac{\sigma^{2}}{\mu_{N}}\cdot \frac{\partial \lambda_{N}}{\partial \mu }\cdot kg\left(k\right)=\frac{\mu }{\mu_{N}}\cdot kg\left(k\right). \end{array}$$

Inserted into the above expression one obtains the ordinary differential equation:

(19) $$\begin{array}{c} \mu_{N}\cdot \left(1-\kappa^{2}t\right)\cdot M_{Z}^{\prime }\left(t\right)=M_{Z}\left(t\right), \end{array}$$

whose unique solution is *M*_*Z*_(*t*) = (1 − *κ* ^2^ *t*)^−*α*^. Since *κ* ^2^ *μ* = *μ*_*Y*_/*α*, one sees that

(20) $$\begin{array}{c} C_{Y}\left(t\right)=\ln M_{Z}\left(\mu t\right)=\alpha \cdot \ln\left\{\frac{\alpha }{\left(\alpha -\mu_{Y}t\right)}\right\} \end{array}$$

is the cgf of a gamma-distributed random variable. The proof is complete.

The proof uses the so-called *natural* parameterization (*p*, *θ*_*N*_, *μ*_*Y*_, *α*) of the compound gamma distribution. It is interesting to obtain explicit parameters orthogonal to the means of *N*, *Y*, and *X*. By the assumption $N\in {\overset{-}{C}}^{⊥}$ one has *μ*_*N*_ = *μ*_*N*_(*p*, *θ*_*N*_)⊥*θ*_*N*_, and since *Y* is gamma distributed, one has $Y\in {\overset{-}{C}}^{⊥}$ with *μ*_*Y*_⊥*α*. It remains to construct a parameter vector orthogonal to the mean of *X* such that

(21) $$\begin{array}{c} \mu =\mu_{N}\left(p,\theta_{N}\right)\cdot \mu_{Y}⊥\left(\theta_{N},\alpha ,\theta \right), \end{array}$$

where *θ* = *θ*(*p*, *θ*_*N*_, *μ*_*Y*_, *α*) must be determined. This task can be solved in a unified way for a lot of counting distributions (see [18, Section 4]). To illustrate the method, it suffices to consider here a single example.

**Example 9 (compound negative binomial gamma distribution)**

Let *N* ~ *NB*(*θ*_*N*_, *p*), *θ*_*N*_ > 0, *p* ∈ (0,1), be a negative binomial random variable. Its cumulant pgf (6) reads *G*(*s*) = −*θ*_*N*_ · ln⁡(1 − *p* · *s*), *λ*_*N*_ = −*θ*_*N*_ · ln⁡(1 − *p*). One has the following identity (see [18, equation (4.7)]):

(22) $$\begin{array}{c} p\frac{\partial G\left(s\right)}{\partial p}=s\cdot G^{\prime }\left(s\right), \end{array}$$

which implies for *s* = 1 that *p*(∂*λ*_*N*_/∂*p*) = *μ*_*N*_. Together, this shows that (10) is satisfied. Therefore, one has $N\in {\overset{-}{C}}^{⊥}$ and *μ*_*N*_ = *θ*_*N*_ · *p*/(1 − *p*)⊥*θ*_*N*_. Now, by Theorem 5 the compound negative binomial will be a compound negative binomial gamma if $X\in {\overset{-}{C}}^{⊥}$ and *σ* ^2^ · (∂*λ*_*N*_/∂*μ*) = *μ* is satisfied. Written in terms of the parameter *α* = (*μ*_*N*_ *κ* ^2^)^−1^, *κ* = *σ*/*μ*, the latter equation is equivalent to the condition (1/*μ*_*N*_)·(∂*λ*_*N*_/∂*μ*) = *α*/*μ*. With ∂*λ*_*N*_/∂*μ* = (∂*λ*_*N*_/∂*p*)·(∂*p*/∂*μ*) = (*μ*_*N*_/*p*)·(∂*p*/∂*μ*) one obtains the differential equation (1/*p*)·(∂*p*/∂*μ*) = *α*/*μ*, which has the solution *p* = *θ* · *μ* ^*α*^ for some *θ*. In the coordinates (*p*, *θ*_*N*_, *μ*_*Y*_, *α*), this constant is equal to

(23) $$\begin{array}{c} \theta =\theta \left(p,\theta_{N},\mu_{Y},\alpha \right)=p\cdot \mu^{-\alpha }=p\cdot {\left(\mu_{Y}\theta_{N}\frac{p}{1-p}\right)}^{-\alpha }. \end{array}$$

Since $X\in {\overset{-}{C}}^{⊥}$ one must have *μ* = *μ*_*N*_(*p*, *θ*_*N*_) · *μ*_*Y*_⊥(*θ*_*N*_, *α*, *θ*).

## 4. Mean Orthogonal Characterization of the Compound Multiparameter Hermite Gamma

The mean orthogonal characterization of the compound gamma distribution allows for a wide variety of count data distributions in the mean orthogonal class. In order to reduce further the possible set of count distributions that can be used, one can ask for characterizations in terms of additional assumptions. For example, Puig [25] and Puig and Valero [26] characterize count data distributions satisfying Gauss's principle and several notions of additivity, which via Theorem 5 can be translated to characterizations of compound gamma distributions. Based on a result by Puig and Valero [20], we derive a most stringent characterization, which allows compounding of the gamma distribution under a single count data family, namely, the multiparameter Hermite distribution. To show this, some additional preliminaries are required.

**Definition 10**

Let *N* be a counting random variable, let *B*_1_(*p*), *B*_2_(*p*),…, be independent and identically distributed Bernoulli random variables with probability of success *p* ∈ (0,1], and let *N* be independent of *B*_*i*_(*p*), *i* = 1,2,…. Then *N*(*p*) = ∑_*i*=1_^*N*^ *B*_*i*_(*p*) (*X*(*p*) = 0  if  *N* = 0) is called an *independent p-thinning* of *N*.

**Definition 11**

Let **F** be a family of count distributions. It is called closed under *binomial subsampling* if, for any random variable *N* with distribution in **F**, all its independent p-thinnings, for all *p* ∈ (0,1], have distributions in **F**.

**Definition 12**

Let **F** be a family of distributions. It is called closed under *convolution* if, for any two independent random variables *X*, *Y* with distributions in **F**, the distribution of the sum *X* + *Y* also belongs to **F**.

**Definition 13**

Let *N* be an integer random variable with pgf *P*(*s*) and *factorial cumulant generating function* (fcgf) *C*_*N*_^*f*^(*s*) = ln⁡*P*(*s* + 1). For any integer *n* ≥ 1 the *n*th *factorial cumulant* of *N* is defined and denoted by *κ*_(*n*)_ = *d* ^*n*^ *C*_*N*_^*f*^(*s*)/*ds* ^*n*^|_*s*=0_.

There is only one count distribution family closed under convolution and binomial subsampling.

**Theorem 14 (Characterization of the multiparameter Hermite distribution)**

Let **F** be a family of count distributions parameterized by its *r* first factorial cumulants *θ* = (*κ*_(1)_,…, *κ*_(*r*)_) and assume that its pgf *P*(*s*) is continuous in *θ* over its parameter space. Then **F** is closed under convolution and binomial subsampling if and only if the pgf is of the form

(24) $$\begin{array}{c} P\left(s\right)=\exp\left\{\sum_{k=1}^{r}\frac{\kappa_{\left(k\right)}}{k!}{\left(s-1\right)}^{k}\right\}. \end{array}$$

**Proof**

See Puig and Valero [20], proof of Theorem 1.

Some comments are in order. The case *r* = 1 corresponds to the Poisson distribution, *r* = 2 is the Hermite distribution (e.g., [27]). For arbitrary *r*, this distribution is called the *multiparameter Hermite* distribution of order *r* by Milne and Westcott [28]. In terms of the cumulant pgf (6), the representation (24) can be rewritten as

(25) $$\begin{array}{c} P\left(s\right)=\exp\left\{\sum_{k=1}^{r}g\left(k\right)\cdot \left(s^{k}-1\right)\right\}, \end{array}$$

where *g*(*k*), *k* = 1,2,…, *r*, solves the system in (8), that is,

(26) $$\begin{array}{c} n\cdot p\left(n\right)=\sum_{k=1}^{\min\left(r,k\right)}k\cdot g\left(k\right)\cdot p\left(n-k\right),\;n=1,2,\ldots ,\\ \sum_{k=1}^{r}g\left(k\right)=-\ln p\left(0\right). \end{array}$$

The case *r* = 2 of (26) is already in A.W. Kemp and C.D. Kemp [29], and for arbitrary *r* this assertion is equivalent to Lemma 2 in Puig and Valero [20]. The special case *g*(1) > 0, *g*(*r*) ≥ 0, *g*(*k*) = 0, *k* = 2,…, *r* − 1 is the generalized Hermite by Gupta and Jain [30]. The multiparameter Hermite belongs also to the Kumar [31] family of distributions. In general, the conditions on the sequence *g*(*k*), *k* = 1,2,…, *r*, under which (25) defines a true probability distribution have been identified in Lévy [32]. According to Lukacs [33, page 252] and Johnson et al. [34, page 356], this is the case provided that a negative value *g*(*k*) < 0 is preceded by a positive value and followed by at least two positive values. In particular, if at least *g*(1), *g*(*r* − 1), *g*(*r*) are nonzero, then *g*(1) > 0, *g*(*r* − 1) > 0, *g*(*r*) > 0, are necessary conditions for (25) to be a pgf [28, Remark 1]. If *g*(*k*) ≥ 0 for *k* = 1,2,…, *r*, then the multiparameter Hermite is compound Poisson with parameter *λ*_*N*_ = ∑_*k*=1_^*r*^ *g*(*k*) and severity *h*(*k*) = *g*(*k*)/*λ*_*N*_, thus infinitely divisible by Feller [35, Section XII.2]. Due to the next result, the multiparameter Hermite is of interest in the context of Gauss's principle, orthogonal parameters to the mean, and the related compound gamma characterization of random sums.

**Lemma 15**

Let *c*_*k*_(*θ*), *k* = 1,2,…, *r*, be continuous real functions in the parameter vector *θ* over some parameter space, and set *g*(*k*) = *c*_*k*_(*θ*) · *p* ^*k*^, *k* = 1,2,…, *r*, for a parameter *p* > 0. Assume that the cumulant pgf *G*(*s*) = ∑_*k*=1_^*r*^ *c*_*k*_(*θ*)·(*ps*)^*k*^ defines a feasible multiparameter Hermite random variable *N* of order *r* over the parameter space. Then $N\in {\overset{-}{C}}^{⊥}$ and *μ*_*N*_ = *μ*_*N*_(*p*, *θ*) = ∑_*k*=1_^*r*^ *k* · *c*_*k*_(*θ*) · *p* ^*k*^⊥*θ*.

**Proof**

Set *λ*_*N*_ = *G*(1) = ∑_*k*=1_^*r*^ *c*_*k*_(*θ*) · *p* ^*k*^. Then one has

(27) $$\begin{array}{c} p\cdot \frac{\partial G\left(s\right)}{\partial p}=s\cdot G^{\prime }\left(s\right),\;\;p\cdot \frac{\partial \lambda_{N}}{\partial p}=\mu_{N}. \end{array}$$

Together, this shows that (10) is satisfied. The result follows by Lemma 7.

**Example 16 (Hermite distribution (*r* = 2))**

Suppose the Hermite distribution is parameterized by its first two factorial cumulants *κ*_(1)_, *κ*_(2)_. Since *κ*_(1)_ = *μ*_*N*_, *κ*_(2)_ = *σ*_*N*_^2^ − *μ*_*N*_, it can equivalently be parameterized by its mean *μ*_*N*_ and variance *σ*_*N*_^2^. Consider a parameterization *p* > 0, *θ*_*N*_ > 0 such that *g*(*k*) = *θ*_*N*_ · *p* ^*k*^, *k* = 1,2. There exists a one-to-one mapping between (*p*, *θ*_*N*_) and (*μ*_*N*_, *θ*_*N*_). Since *μ*_*N*_ = *g*(1) + 2*g*(2),*σ*_*N*_^2^ = *g*(1) + 4*g*(2), it is determined by the coordinate transformation:

(28) $$\begin{array}{c} p=\frac{1}{2}\cdot \frac{\sigma_{N}^{2}-\mu_{N}}{\sigma_{N}^{2}+2\mu_{N}},\;\;\theta_{N}=2\cdot \frac{{\left(\sigma_{N}^{2}+2\mu_{N}\right)}^{2}}{\sigma_{N}^{2}-\mu_{N}}. \end{array}$$

Therefore, the cumulant pgf *G*(*s*) = *θ* · (*ps* + *p* ^2^ *s* ^2^) defines a feasible two-parameter Hermite distribution such that the corresponding random variable belongs to ${\overset{-}{C}}^{⊥}$ and *μ*_*N*_ = *μ*_*N*_(*p*, *θ*_*N*_) = *θ*_*N*_ · *p* · (1 + 2*p*)⊥*θ*_*N*_. Since *σ*_*N*_^2^ > *μ*_*N*_ one notes that the Hermite distribution is necessarily overdispersed. As noted by Puig and Valero [20] overdispersion holds for all infinitely divisible multiparameter Hermite distributions of arbitrary order *r* ≥ 2. Therefore, it should be useful to analyze data with this property (e.g., claim number data in automobile insurance, up-to-date Hürlimann [36, page 802], and multiparameter Hermite for *r* ≥ 3).

We are ready for the following new characterization result.

**Theorem 17 (Compound multiparameter Hermite gamma characterization)**

Let *N* be a counting random variable parameterized by its *r* first factorial cumulants *ξ* = (*κ*_(1)_,…, *κ*_(*r*)_) and assume that its cgf *C*_*N*_(*t*; *ξ*) is continuous in *ξ* over its parameter space and set *μ*_*N*_ = *κ*_(1)_, *λ*_*N*_ = −ln Pr(*N* = 0). Suppose the cgf *C*_*Y*_(*t*) of the severity *Y* exists, and let *C*(*t*) = *C*_*N*_(*C*_*Y*_(*t*)) be the cgf of the random sum *X* = ∑_*i*=1_^*N*^ *Y*_*i*_. Assume the cgf of the mean scaled severity *C*_*Z*_(*t*) = *C*_*Y*_(*t*/*μ*) is functionally independent of *μ*, and set *α* = (*μ*_*N*_ *κ* ^2^)^−1^, *κ* = *σ*/*μ*. Assume *N* is closed under convolution and binomial subsampling, $X\in {\overset{-}{C}}^{⊥}$ and *σ* ^2^ · (∂*λ*_*N*_/∂*μ*) = *μ*. Then *N* is a multiparameter Hermite distribution of order *r*, and *Y* is gamma distributed with cgf *C*_*Y*_(*t*) = *α* · ln⁡{*α*/(*α* − *μ*_*Y*_ *t*)}. Furthermore, there exists a parameterization (*p*, *θ*_*N*_) of N such that its cumulant pgf reads *G*(*s*) = ∑_*k*=1_^*r*^ *c*_*k*_(*θ*_*N*_)·(*ps*)^*k*^. One has $N,Y,X\in {\overset{-}{C}}^{⊥}$ with *μ*_*N*_ = *μ*_*N*_(*p*, *θ*_*N*_) = ∑_*k*=1_^*r*^ *k* · *c*_*k*_(*θ*_*N*_) · *p* ^*k*^⊥*θ*_*N*_, *μ*_*Y*_⊥*α*, *μ* = *μ*_*N*_(*p*, *θ*_*N*_) · *μ*_*Y*_⊥(*θ*_*N*_, *α*, *θ*), and, in the coordinates (*p*, *θ*_*N*_, *μ*_*Y*_, *α*), the constant *θ* is equal to

(29) θ=θ(p,θN,μY,α)=p·μ−α=p·(μY·∑k=1rk·ck(θN)·pk)−α.

**Proof**

The result follows by combining Theorems 5 and 14 making the observation that a multiparameter Hermite distribution can always be put in the form of Lemma 15 (generalization of Example 16). The assertion about the orthogonal parameters to the means *μ*_*N*_, *μ*_*Y*_, *μ* follows along the same arguments as in Example 9 using (27).
